# Supplementary material for: Systemic Antimicrobial Treatment of Chronic Osteomyelitis in Adults: A Narrative Review
Source: Antibiotics (Basel). 2023 May 23;12(6):944. doi: 10.3390/antibiotics12060944 (PMC10294961; doi:10.3390/antibiotics12060944)
Supplement: Supplementary file 1 [file antibiotics-12-00944-s001.zip › antibiotics-2402123-supplementary.pdf]

Supplementary material File S1

PICO strategy (Patient/Problem, Intervention, Comparison, Outcome)

1. PICO
   P: adult patients with chronic osteomyelitis
   I: short-term antibiotic therapy (< 6 weeks)
   C: long-term antibiotic therapy (≥ 6 weeks)
   O: mortality, relapse rate, adverse effects
2. PICO
   P: adult patients with chronic osteomyelitis
   I: oral antibiotic therapy or short-term parenteral therapy (< 2 weeks) followed by oral antibiotic therapy
   C: parenteral antibiotic therapy
   O: mortality, relapse rate, adverse effects
3. PICO
   P: adult patients with chronic osteomyelitis
   I: combination antibiotic therapy with rifampicin for staphylococcal infections
   C: antibiotic therapy without addition of rifampicin
   O: mortality, relapse rate, adverse effects

Search strategy for PUBMED

PICO 1

("osteomyelitis"[MeSH Terms] OR "osteomyelitis*"[Title/Abstract] OR "spondylitis"[MeSH Terms] OR "spondylitis*"[Title/Abstract] OR "osteitis"[MeSH Terms] OR "osteitis"[Title/Abstract] OR "bone infection*"[Title/Abstract]) AND ("treatment outcome"[MeSH Terms] OR "treatment outcome*"[Title/Abstract] OR "efficac*"[Title/Abstract] OR "effectiveness*"[Title/Abstract] OR "recurrence"[MeSH Terms] OR "recurrence*"[Title/Abstract] OR "relapse*"[Title/Abstract] OR "reinfection"[MeSH Terms] OR "reinfection*"[Title/Abstract] OR "recurrent infection*"[Title/Abstract] OR "symptom flare up"[MeSH Terms] OR "symptom flare up*"[Title/Abstract] OR "symptom exacerbation*"[Title/Abstract] OR "mortality"[MeSH Terms] OR "mortalit*"[Title/Abstract] OR "fatality rate*"[Title/Abstract] OR "death rate*"[Title/Abstract] OR "drug related side effects and adverse reactions"[MeSH Terms] OR "drug related side effects and adverse reaction*"[Title/Abstract] OR "adverse reaction*"[Title/Abstract] OR "adverse drug reaction*"[Title/Abstract] OR "side effect*"[Title/Abstract] OR "adverse event*"[Title/Abstract] OR "adverse drug event*"[Title/Abstract] OR "drug toxicity"[Title/Abstract]) AND ("anti-bacterial agents"[MeSH Terms] OR "antibacterial*"[Title/Abstract] OR "anti bacterial*"[Title/Abstract] OR "antibiotic*"[Title/Abstract] OR "anti-infective agents"[MeSH Terms] OR "anti infective*"[Title/Abstract] OR "antiinfective*"[Title/Abstract] OR "anti microbial*"[Title/Abstract] OR "antimicrobial*"[Title/Abstract] OR "anti microbial"[Title/Abstract]) AND ("duration of therapy"[MeSH Terms] OR "duration of therapy"[Title/Abstract] OR "therapy duration*"[Title/Abstract] OR "duration of treatment"[Title/Abstract] OR "treatment duration*"[Title/Abstract] OR "antibiotic duration*"[Title/Abstract] OR "duration of antibiotic*"[Title/Abstract] OR "duration of antimicrobial"[Title/Abstract] OR "antimicrobial duration*"[Title/Abstract] OR "treatment length"[Title/Abstract] OR "length of therapy"[Title/Abstract] OR "length of treatment"[Title/Abstract] OR "therapy

length"[Title/Abstract])

PICO 2

("osteomyelitis"[MeSH Terms] OR "osteomyelitis*"[Title/Abstract] OR "spondylitis"[MeSH Terms] OR "spondylitis*"[Title/Abstract] OR "osteitis"[MeSH Terms] OR "osteitis"[Title/Abstract] OR "bone infection*"[Title/Abstract]) AND ("treatment outcome"[MeSH Terms] OR "treatment outcome*"[Title/Abstract] OR "efficac*"[Title/Abstract] OR "effectiveness*"[Title/Abstract] OR "recurrence"[MeSH Terms] OR "recurrence*"[Title/Abstract] OR "relapse*"[Title/Abstract] OR "reinfection"[MeSH Terms] OR "reinfection*"[Title/Abstract] OR "recurrent infection*"[Title/Abstract] OR "symptom flare up"[MeSH Terms] OR "symptom flare up*"[Title/Abstract] OR "symptom exacerbation*"[Title/Abstract] OR "mortality"[MeSH Terms] OR "mortalit*"[Title/Abstract] OR "fatality rate*"[Title/Abstract] OR "death rate*"[Title/Abstract] OR "drug related side effects and adverse reactions"[MeSH Terms] OR "drug related side effects and adverse reaction*"[Title/Abstract] OR "adverse reaction*"[Title/Abstract] OR "adverse drug reaction*"[Title/Abstract] OR "side effect*"[Title/Abstract] OR "adverse event*"[Title/Abstract] OR "adverse drug event*"[Title/Abstract] OR "drug toxicity"[Title/Abstract]) AND ("anti-bacterial agents"[MeSH Terms] OR "antibacterial*"[Title/Abstract] OR "anti bacterial*"[Title/Abstract] OR "antibiotic*"[Title/Abstract] OR "anti-infective agents"[MeSH Terms] OR "anti infective*"[Title/Abstract] OR "antiinfective*"[Title/Abstract] OR "anti microbial*"[Title/Abstract] OR "antimicrobial*"[Title/Abstract] OR "anti microbial"[Title/Abstract]) AND ("Drug Administration Routes"[MeSH Terms] OR "drug administration route*"[Title/Abstract] OR "route of administration*"[Title/Abstract] OR "administration, oral"[MeSH Terms] OR "oral*"[Title/Abstract] OR "peroral*"[Title/Abstract] OR "injections, intravenous"[MeSH Terms] OR "infusions, intravenous"[MeSH Terms] OR "infusions, parenteral"[MeSH Terms] OR "intravenous*"[Title/Abstract] OR "parenteral*"[Title/Abstract] OR "infusion*"[Title/Abstract] OR "per os*"[Title/Abstract])

PICO 3

("osteomyelitis"[MeSH Terms] OR "osteomyelitis*"[Title/Abstract] OR "spondylitis"[MeSH Terms] OR "spondylitis*"[Title/Abstract] OR "osteitis"[MeSH Terms] OR "osteitis"[Title/Abstract] OR "bone infection*"[Title/Abstract]) AND ("treatment outcome"[MeSH Terms] OR "treatment outcome*"[Title/Abstract] OR "efficac*"[Title/Abstract] OR "effectiveness*"[Title/Abstract] OR "recurrence"[MeSH Terms] OR "recurrence*"[Title/Abstract] OR "relapse*"[Title/Abstract] OR "reinfection"[MeSH Terms] OR "reinfection*"[Title/Abstract] OR "recurrent infection*"[Title/Abstract] OR "symptom flare up"[MeSH Terms] OR "symptom flare up*"[Title/Abstract] OR "symptom exacerbation*"[Title/Abstract] OR "mortality"[MeSH Terms] OR "mortalit*"[Title/Abstract] OR "fatality rate*"[Title/Abstract] OR "death rate*"[Title/Abstract] OR "drug related side effects and adverse reactions"[MeSH Terms] OR "drug related side effects and adverse reaction*"[Title/Abstract] OR "adverse reaction*"[Title/Abstract] OR "adverse drug reaction*"[Title/Abstract] OR "side effect*"[Title/Abstract] OR "adverse event*"[Title/Abstract] OR "adverse drug event*"[Title/Abstract] OR "drug toxicity"[Title/Abstract]) AND ("anti-bacterial agents"[MeSH Terms] OR "antibacterial*"[Title/Abstract] OR "anti bacterial*"[Title/Abstract] OR "antibiotic*"[Title/Abstract] OR "anti-infective agents"[MeSH Terms] OR "anti infective*"[Title/Abstract] OR "antiinfective*"[Title/Abstract] OR "anti microbial*"[Title/Abstract] OR "antimicrobial*"[Title/Abstract] OR "anti microbial"[Title/Abstract]) AND ("rifampin"[MeSH Terms] OR "rifampin*"[Title/Abstract] OR "rifampicin*"[Title/Abstract] OR ("Staphylococcal

Infections"[MeSH Terms] OR "staphylococcal*"[Title/Abstract]) OR ("staphylococcus"[MeSH Terms] OR "staphylococcus*"[Title/Abstract]))

Search strategy for OVID-EMBASE

#	Query	Results from 5 Jan 2023
1	exp osteomyelitis/	44,714
2	osteomyelitis$.ti,ab.	28,690
3	exp spondylitis/	76,056
4	spondylitis$.ti,ab.	29,531
5	exp osteitis/	63,821
6	osteitis$.ti,ab.	5,092
7	exp bone infection/	11,600
8	bone infection$.ti,ab.	2,189
9	1 or 2 or 3 or 4 or 5 or 6 or 7 or 8	150,364
10	exp treatment outcome/	2,122,926
11	treatment outcome$.ti,ab.	92,859
12	exp recurrent disease/	205,878
13	recurrent disease$.ti,ab.	23,699
14	recurrence$.ti,ab.	564,094
15	exp relapse/	169,369
16	relapse$.ti,ab.	334,619
17	exp reinfection/	33,284
18	reinfection$.ti,ab.	12,722
19	symptom flare up$.ti,ab.	35
20	exp disease exacerbation/	169,755
21	exacerbation$.ti,ab.	103,428
22	exp mortality/	1,304,932
23	mortalit$.ti,ab.	1,381,734
24	fatality rate$.ti,ab.	16,262
25	death rate$.ti,ab.	32,560
26	exp side effect/	644,716
27	side effect$.ti,ab.	424,426
28	exp adverse event/	745,673
29	adverse event$.ti,ab.	371,941
30	adverse drug reaction$.ti,ab.	30,430
31	adverse reaction$.ti,ab.	61,688
32	exp drug toxicity/	149,329
33	drug toxicit$.ti,ab.	9,427
34	10 or 11 or 12 or 13 or 14 or 15 or 16 or 17 or 18 or 19 or 20 or 21 or 22 or 23 or 24 or 25 or 26 or 27 or 28 or 29 or 30 or 31 or 32 or 33	5,744,220

35	exp antiinfective agent/	4,309,752
36	antimicrobial$.ti,ab.	265,587
37	antibacterial$.ti,ab.	122,860
38	antibiotic$.ti,ab.	516,723
39	antiinfective$.ti,ab.	1,126
40	anti microbial$.ti,ab.	8,680
41	anti bacterial$.ti,ab.	6,968
42	anti infective$.ti,ab.	7,637
43	exp antibiotic therapy/	147,347
44	antibiotic therap$.ti,ab.	53,135
45	antibiotic treatment$.ti,ab.	39,109
46	anti biotic$.ti,ab.	394
47	35 or 36 or 37 or 38 or 39 or 40 or 41 or 42 or 43 or 44 or 45 or 46	4,499,479
48	exp treatment duration/	280,011
49	treatment duration$.ti,ab.	27,576
50	duration of treatment$.ti,ab.	26,356
51	therapy duration$.ti,ab.	3,001
52	duration of therap$.ti,ab.	11,665
53	treatment length$.ti,ab.	1,697
54	length of treatment$.ti,ab.	3,871
55	therapy length$.ti,ab.	398
56	length of therap$.ti,ab.	1,119
57	antibiotic duration$.ti,ab.	779
58	duration of antibiotic$.ti,ab.	3,668
59	antimicrobial duration$.ti,ab.	91
60	duration of antimicrobial$.ti,ab.	771
61	48 or 49 or 50 or 51 or 52 or 53 or 54 or 55 or 56 or 57 or 58 or 59 or 60	312,119
62	exp drug administration route/	1,159,607
63	administration route$.ti,ab.	5,509
64	route of administration$.ti,ab.	14,783
65	exp oral drug administration/	393,550
66	oral$.ti,ab.	1,052,968
67	peroral$.ti,ab.	8,635
68	per os$.ti,ab.	8,293
69	exp parenteral drug administration/	754,200
70	parenteral$.ti,ab.	80,796
71	intravenous$.ti,ab.	499,591
72	infusion$.ti,ab.	371,906
73	62 or 63 or 64 or 65 or 66 or 67 or 68 or 69 or 70 or 71 or 72	2,657,717
74	exp rifampicin/	97,180
75	rifampicin$.ti,ab.	23,883
76	rifampin$.ti,ab.	11,408

77	exp Staphylococcus/	248,510
78	staphylococcus$.ti,ab.	173,306
79	exp Staphylococcus infection/	50,910
80	staphylococcal$.ti,ab.	27,603
81	74 or 75 or 76 or 77 or 78 or 79 or 80	388,210
82	9 and 34 and 47	15,096
83	61 and 82	1,986
84	73 and 82	3,286
85	81 and 82	4,808

exp osteomyelitis/
osteomyelitis$.ti,ab.
exp spondylitis/
spondylitis$.ti,ab.
exp osteitis/
osteitis$.ti,ab.
exp bone infection/
bone infection$.ti,ab.
1 or 2 or 3 or 4 or 5 or 6 or 7 or 8
exp treatment outcome/
treatment outcome$.ti,ab.
exp recurrent disease/
recurrent disease$.ti,ab.
recurrence$.ti,ab.
exp relapse/
relapse$.ti,ab.
exp reinfection/
reinfection$.ti,ab.
symptom flare up$.ti,ab.
exp disease exacerbation/
exacerbation$.ti,ab.
exp mortality/
mortalit$.ti,ab.
fatality rate$.ti,ab.
death rate$.ti,ab.
exp side effect/
side effect$.ti,ab.
exp adverse event/
adverse event$.ti,ab.
adverse drug reaction$.ti,ab.
adverse reaction$.ti,ab.
exp drug toxicity/
drug toxicit$.ti,ab.
10 or 11 or 12 or 13 or 14 or 15 or 16 or 17 or 18 or 19 or 20 or 21 or 22 or 23 or 24 or 25 or 26 or 27

or 28 or 29 or 30 or 31 or 32 or 33
exp antiinfective agent/
antimicrobial$.ti,ab.
antibacterial$.ti,ab.
antibiotic$.ti,ab.
antiinfective$.ti,ab.
anti microbial$.ti,ab.
anti bacterial$.ti,ab.
anti infective$.ti,ab.
exp antibiotic therapy/
antibiotic therap$.ti,ab.
antibiotic treatment$.ti,ab.
anti biotic$.ti,ab.
35 or 36 or 37 or 38 or 39 or 40 or 41 or 42 or 43 or 44 or 45 or 46
exp treatment duration/
treatment duration$.ti,ab.
duration of treatment$.ti,ab.
therapy duration$.ti,ab.
duration of therap$.ti,ab.
treatment length$.ti,ab.
length of treatment$.ti,ab.
therapy length$.ti,ab.
length of therap$.ti,ab.
antibiotic duration$.ti,ab.
duration of antibiotic$.ti,ab.
antimicrobial duration$.ti,ab.
duration of antimicrobial$.ti,ab.
48 or 49 or 50 or 51 or 52 or 53 or 54 or 55 or 56 or 57 or 58 or 59 or 60
exp drug administration route/
administration route$.ti,ab.
route of administration$.ti,ab.
exp oral drug administration/
oral$.ti,ab.
peroral$.ti,ab.
per os$.ti,ab.
exp parenteral drug administration/
parenteral$.ti,ab.
intravenous$.ti,ab.
infusion$.ti,ab.
62 or 63 or 64 or 65 or 66 or 67 or 68 or 69 or 70 or 71 or 72
exp rifampicin/
rifampicin$.ti,ab.
rifampin$.ti,ab.
exp Staphylococcus/
staphylococcus$.ti,ab.

exp Staphylococcus infection/
staphylococcal$.ti,ab.
74 or 75 or 76 or 77 or 78 or 79 or 80
9 and 34 and 47
61 and 82
73 and 82
81 and 82
